# Enhanced neutralizing antibody response induced by inactivated enterovirus 71 in cynomolgus monkeys

**DOI:** 10.1371/journal.pone.0202552

**Published:** 2018-10-02

**Authors:** Hyun Ju In, Heeji Lim, Jung-Ah Lee, Sang-Rae Lee, Yeung Bae Jin, Kang-Jin Jeong, Ji-Yeon Hyeon, Jung Sik Yoo, June-Woo Lee, Young Ki Choi, Sang-Won Lee

**Affiliations:** 1 Division of Vaccine Research, National Research Institute of Health, Centers for Disease Control and Prevention, Osong-eup, CheongJu, Chungcheongbuk-do, Republic of Korea; 2 College of Medicine and Medical Research Institute, Chungbuk National University, CheongJu, Chungcheongbuk-do, Republic of Korea; 3 The National Primate Research Center, Korea Research Institute of Bioscience and Biotechnology, Ochang-eup, CheongJu, Chungcheongbuk-do, Republic of Korea; Instituto Butantan, BRAZIL

## Abstract

Enterovirus 71 (EV71) is a major etiological agent of various public health issues, particularly in the Asia-Pacific region. EV71 causes hand-foot-and-mouth disease (HFMD) and is associated with serious neurological disorders in young children. A formalin-inactivated EV71 candidate vaccine (KCDC-HFMDV1-EV71) based on the C4 subgenotype was previously developed and confirmed to be a potential candidate vaccine for prevention of EV71 infection in mice. In this study, an inactivated EV71 vaccine was used for analysis of long-term immunogenicity and efficacy in cynomolgus monkeys, a common nonhuman primate model. The vaccine was immunized three times at 0, 4, and 8 weeks with either 20-μg doses of EV71 candidate vaccine formulated with aluminum hydroxide gel adjuvant or phosphate-buffered saline as a control. The group immunized with the inactivated EV71 showed significantly increased EV71-specific antibody and serum neutralizing antibody titers at 3 weeks after vaccination and maintained these elevated titers until the end of the experiment (54 weeks after vaccination). The sera from vaccinated cynomolgus monkeys showed a crossreactive neutralizing antibody response to the heterologous subtype of EV71 (B1–4, C1, and C2). These findings suggest that the inactivated EV71 candidate vaccine may be a potential vaccine candidate and valuable tool for the control of HFMD.

## Introduction

Enterovirus 71 (EV71) infection has emerged as a serious threat to public health in young children worldwide [1]. EV71 belongs to the genus *Enterovirus*, family *Picornaviridae*. EV71 is a nonenveloped, single-stranded, positive-sense RNA virus and is classified into four genotypes, i.e., genotypes A, B, C, and D, based on the *VP1* gene sequence. Genotypes A and D are represented by single strains, i.e., BrCr and India strains, respectively, whereas genotypes B and C were each divided five subgenotypes, designated B1–B5 and C1–C5. Genotypes B and C are distributed worldwide, whereas genotype A is much less common [2, 3]. Recombination events between genotypes of EV71 have generated a new subgenotype [4].

EV71 was identified as a major causative agent of hand-foot-and-mouth disease (HFMD) in 1969, when the virus was first isolated [5]. Outbreaks of EV71 infection have been reported frequently in the Asia-Pacific region, and the infection is associated with neurological disorders in children [6–8]. More than 80% of patients who die from HFMD with neurological symptom show EV71 infection [7, 9]. Diverse subgenotypes have been reported in HFMD outbreaks in Malaysia (B3 in 1997, B4 in 2000), Taiwan (C2 in 1998, B4 in 2000), Singapore (B4 in 2000), Vietnam (C5 in 2005), China (C4 from 2008), and Korea (C3 in 2000, C4 in 2009) [10].

The neutralizing antibody response is important for the prevention of EV71 infection and transmission [11]. Moreover, T-cell immune responses play a critical role in controlling the disease [12, 13]. Development of an ideal EV71 vaccine inducing both humoral and cellular immune responses is critical. Several types of EV71 vaccine candidates have been developed, including inactivated virus vaccines, attenuated virus vaccines, peptide-based vaccines, and virus-like particle (VLP) vaccines[14–20]. Among these vaccines, the inactivated EV71 vaccine is the most promising, and clinical trials for several vaccines targeting poliovirus have already being tested [19–23]. Inactivated vaccines are considered ideal owing to concerns with the potential for vaccine-derived virulent viruses, e.g., from poliovirus attenuated vaccine [24–26]. In particular, our group recently showed that formalin-inactivated HFMD vaccine using an EV71 strain was a potential candidate vaccine for prevention of EV71 infection [27]

EV71 has a limited host range; only humans can be infected. Thus, a major challenge in EV71 vaccine development is the lack of an animal model for efficacy tests. For preclinical trials of vaccines, animal experiments with mice or nonhuman primates (NHPs) are required. NHP models are more suitable for evaluating the immunogenicity and efficacy of vaccine candidates than mouse models [28] because adult mice are resistant to EV71 infection and suckling mice are hard to manage.

Therefore, in this study, we evaluated the efficacy of the inactivated EV71 vaccine in NHPs, allowing us to analyze potent and long-lasting humoral immune responses. The aim of the present study was to investigate the efficacy of the inactivated EV71 vaccine and to establish a basis for further studies of the protective capacity of the vaccine by challenge with EV71 in NHPs.

## Materials and methods

### Ethics statement

This study was designed and approved by Korea National Institutes of Health (Experimental approval number: KCDC-024-16-2A). All animal experiments were conducted in strict accordance with the Guidelines of the Institutional Animal Care and Use Committee (KRIBB-AEC-14126) in the Korea Research Institute of Bioscience and Biotechnology (KRIBB).

### Animals

The monkeys used in the experiment were purchased through formal import procedures from Zhaoqing Laboratory Animal Research Center (Guangdong Province, China) with the Convention on International Trade in Endangered Species of Wild Fauna and Flora (CITES) permit. All animals were maintained at the National Primate Research Center (NPRC) in Korea Research Institute of Bioscience and Biotechnology (KRIBB). For this study, monkeys were moved to individual indoor cages designed to allow the monkeys to contact each other in vision but avoiding physical touch to prevent contamination. The dimensions of the cages were 60ⅹ80ⅹ80cm as per the accordance with the Guide for the Care and Use of Laboratory Animal: Eighth Edition (National Research Council, 2011). To enrich the environment, water, monkey chow (Teklad 2050, Harlan, USA), and various fresh fruits including apples, bananas, and grapes were supplied *ad libitum*. The perches were installed in individual cages. Each primates was given various rubber and plastic toys. If monkeys did not show interest in those toys, toys of different shapes and sizes were put inside the cages. Environmental conditions were controlled with a temperature of 24 ± 2°C, a relative humidity of 50 ± 5%, and a 12:12hr light:dark cycle. The health and behavioral disorders of the monkeys were monitored by animal care staff three times a day. Before and after intervention, the primate veterinarian evaluated vital signs, body weight, clinical signs, and hematological and biochemical values. They were also monitored by microbiological tests including those for B virus, simian retrovirus (SRV), simian immunodeficiency virus (SIV), simian virus 40 (SV40), and simian T-cell lymphotropic virus (STLV), tuberculosis, every year. As all collections of blood samples and vaccine administration were performed under anesthesia, the primates were anesthetized by an intramuscular injection of ketamine (5mg /kg) into the thigh muscle. Blood samples were collected from the femoral vein, and the vaccine was administered to arm muscle. A humane euthanasia procedure was performed by injecting pentobarbital (100 mg/kg) intravenously under the recommendation of the certified veterinarian, if the animal was diagnosed with certain diseases such as unknown fever, and severe diarrhea (containing blood, mucus, and pus), was moribund, or was not eating/drinking and did not show improvement following medication or environmental enrichment.

### Production of inactivated EV71 vaccines

The KCDC-HFMDV1-EV71 vaccine strain (C4a subgenotype) was isolated from a patient with HFMD in Korea in 2012. The strain was propagated, and viral titers were evaluated in Vero cells (green monkey kidney cells) using Dulbecco’s modified Eagle medium (DMEM). Viral titers were determined by microtitration using Vero cells and expressed as the 50% tissue culture infective dose (TCID_50_), according to the Reed-Muench method. The vaccine strain was inactivated using formaldehyde (Sigma-Aldrich, St. Louis, MO, USA) at 37°C for 5 days and mixed with an equal volume of aluminum hydroxide (InvivoGen, San Diego, CA, USA), which is the most commonly used adjuvant in human vaccines.

### Immunization of monkeys

Five cynomolgus monkeys (*Macaca fascicularis*), 3 years of age (body weight of 2.88–3.33 kg), were randomly allocated into two groups (G1 and G2); monkeys in the G1 group (n = 3) and G2 group (n = 2) were intramuscularly administrated with 0.5ml of 20 μg of inactivated EV71 vaccine mixed with 2.5 mg alum or phosphate-buffered saline (PBS), respectively. The monkeys in G1 group were boost vaccinated at 4 or 8 weeks after primary vaccination, using the same vaccine. All animals were provided by the National Primate Research Center (NPRC) of Korea. Body weight and temperature of monkeys were measured individually and recorded. Blood samples were collected (1–2 ml) at 0, 3, 7, 12, 28, and 54 weeks after vaccination. Hematological analyses were conducted using an automated hematology analyzer (CELL_DYN 3700; Abbott, IL, USA) according to the manufacturer’s instructions. All animal experiments were conducted in strict accordance with the Guidelines of the Institutional Animal Care and Use Committee (KRIBB-AEC-14126) in the Korea Research Institute of Bioscience and Biotechnology (KRIBB).

### Serum neutralizing assay

The serum neutralizing (SN) assay was conducted in 96-well microplates containing confluent Vero cells that were seeded 24 h before the experiment. The monkey sera were inactivated at 56°C for 30 min and stored at -80°C prior to use. An equal volume of 2-fold serially diluted serum samples was mixed with 50 μL of 100 TCID_50_ EV71 (A, B1, B2, B3, B4, C1, C2, C3, C4a, or C5 subgenotypes) and incubated at 37°C for 2 h. The mixture was applied to a monolayer of Vero cells, which were subsequently incubated at 37°C for 5 days. The SN titer was determined as the highest dilution that gave no cellular cytopathic effects (CPEs).

### Measurement of EV71-specific antibodies

EV71-specific IgG antibodies and IgG subclasses in monkey serum were measured by enzyme-linked immunosorbent assays (ELISAs). Ninety-six-well microplates were coated with 100 ng/100 μL of EV71 antigen, and subsequently incubated overnight at 4°C. After blocking with 100 μL of PBST (PBS containing 0.1% Tween-20) containing 5% skim milk at 37°C for 1 h, 100 μL of 1:200 test sera in dilution buffer (5% /(w/v)) skim milk in PBST) was added to each well. After incubation for 1 h, the plates were washed, and a 100-μL aliquot of 1:1000 rabbit anti-monkey IgG diluted in dilution buffer was added. The plates were then incubated at 37°C for 1 h, followed by the addition of a 100-μL aliquot of 1:5000 horseradish peroxidase (HRP)-conjugated anti-rabbit as the third antibody. After incubation for 1 h at 37°C, the plates were developed with 100 μL of 3,3’,5,5’-tetra-methylbenzidine (GenDEPOT, Barker, TX, USA) in the dark. The optical density (OD) of the solution was measured at 450 nm. The anti-EV71 IgG subclasses were determined using a human IgG subclass profile kit (Invitrogen Life Technologies, Gaithersburg, MD, USA) according to the manufacturer’s instructions.

### Quantification of cytokine levels using a multiplex system

For cytokine assays, monkey sera were isolated from blood, and cytokine levels were evaluated following administration of the inactivated EV71 vaccine using a Monkey Cytokine Magnetic 29-plex kit (Life Technologies) on a MAGPIX analyzer system, according to the manufacturer’s instructions.

### Statistical analysis

Statistical analyses were performed using GraphPad Prism version 5 or InStat3. Data were analyzed using two-way analysis of variance (ANOVA), followed by Bonferroni post-hoc test. Results with *P* values of less than 0.05 were considered statistically significant.

## Results

### Clinical signs and hematology of monkeys after EV71 vaccination

To determine the safety of the inactivated EV71 vaccine, general clinical observations were evaluated. The monkeys administered the inactivated EV71 vaccine were as healthy as monkeys in the mock group. Body weights and body temperatures were not significantly different among groups (data not shown). Significant changes in the numbers of lymphocytes (measured by changes in white blood cells (WBCs), red blood cells (RBCs), platelets (PLTs), and eosinophils) after vaccination were not observed in either group of monkeys (Table 1). These results indicated that the inactivated EV71 vaccine used in this study was safe.

**Table 1:** Hematologic analysis of the monkeys after vaccination with inactivated EV71 vaccine.

Group	WBC (10^3^/μl)	RBC (10^6^/μl)	PLT (10^6^/μl)	Eosinophil (%)
EV71	9.05±1.52	5.00±0.11	592.67±78.13	6.03±2.01
MOCK	10.06±0.20	5.06±0.21	451.50±36.50	5.32±0.34
Normal index	5.0~21.1	4.60~7.30	259~734	0~10.4

### Immunogenicity of inactivated EV71 vaccine

**Fig 1:**
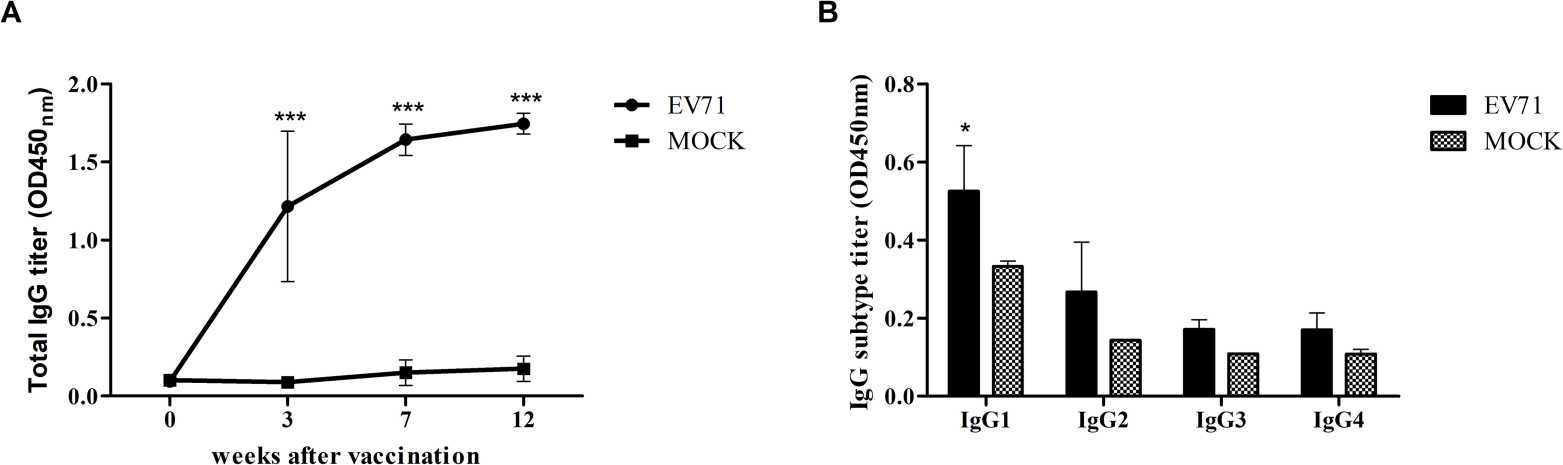
EV71-specific IgG responses induced by the inactivated EV71 vaccine. Cynomolgus monkeys were vaccinated with inactivated EV71 vaccine (EV71) or PBS (mock) as a control. Serology, including EV71-specific total IgG (A) and IgG subclass (B) responses, was assessed by ELISA. ***P < 0.001 using Two-way ANOVA with Bonferroni post-hoc test compared with the control group (mock).

To determine whether the inactivated EV71 vaccine could elicit a humoral immune response in NHPs, five cynomolgus monkeys were inoculated with the vaccine, and its immunogenicity was assessed. Humoral immunity was significantly increased in EV71-vaccinated monkeys as compared with those in the mock group (Fig 1A). EV71-specific IgG subclasses were measured using sera at 12 weeks after vaccination by IgG subclass ELISA. All IgG subclasses (IgG1, IgG2, IgG3, and IgG4) in the vaccinated group were higher than those in the mock group (Fig 1B).

**Fig 2:**
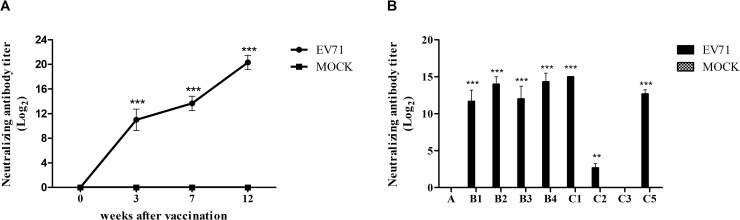
EV71-specific SN antibody titers against homologous and heterologous strains after EV71 vaccination. Cynomolgus monkeys were vaccinated with inactivated EV71 vaccine (EV71) or PBS (mock) as a control. The levels of serum neutralizing antibodies against EV71 were measured using the vaccine strain (A) and nine heterologous strains belonging to different subgenotypes (B). ***P < 0.001 using Two-way ANOVA with Bonferroni post-hoc test.

The SN antibody titers in the vaccinated group began to increase at 3 weeks after vaccination and remained elevated until 12 weeks; these changes were not detected in the mock group (Fig 2A). The SN antibody titers against the heterologous subgenotypes (A, B1, B2, B3, B4, C1, C2, C3, and C5) were measured using sera at 12 weeks after vaccination (Fig 2B). Among the heterologous subgenotypes, crossreactivity was observed against six subgenotypes (B1, B2, B3, B4, C1, and C5) in the inactivated EV71 vaccine group.

**Fig 3:**
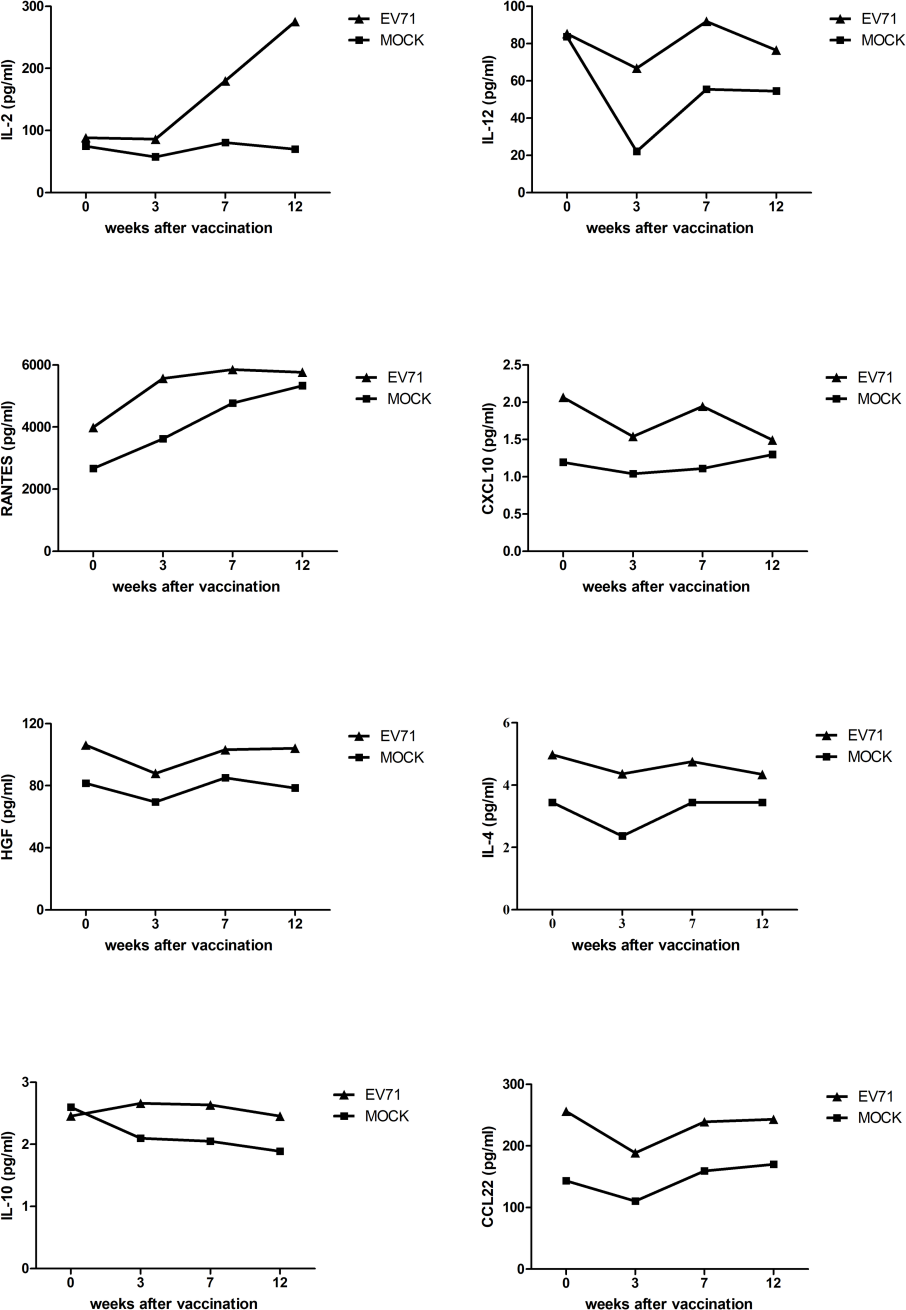
EV71-specific cell-mediated immune responses induced by the inactivated EV71 vaccine. Cynomolgus monkeys were vaccinated with inactivated EV71 vaccine (EV71) or PBS (mock) as a control. PBMCs from monkeys were isolated and stimulated with EV71 vaccine. The supernatants of cultured PBMCs were harvested at 24 h after stimulation and analyzed for cytokines using a multiplex system.

To evaluate cell-mediated immune responses to the inactivated EV71 vaccine, peripheral blood mononuclear cells (PBMCs) from vaccinated monkeys were isolated and used to measure induced cytokines through a multiplex system. Interleukin (IL)-2, IL-12, regulated on activation, normal T cell expressed and secreted (RANTES), C-X-C motif chemokine 10 (CXCL10), hepatocyte growth factor (HGF), IL-4, IL-10, and C-C motif chemokine 22 (CCL22) were induced in vaccinated monkeys compared with those in unvaccinated monkeys (Fig 3). In particular, vaccinated monkeys showed higher levels of IL-2 and IL-12, which are involved in the Th1 immune response, and CCL22, which is involved in the Th2 immune response. These results indicated that the inactivated EV71 vaccine triggered mixed Th1/Th2 immune responses in NHPs. In particular, the Th1 immune response was induced more strongly than the Th2 immune response by the vaccine candidate.

### Immunogenicity of the inactivated EV71 vaccine in a 1-year study

To investigate maintenance of humoral immune responses against the inactivated EV71 vaccine over a 1-year period, serum samples from monkeys were collected at 28 and 54 weeks after vaccination and tested by ELISA and SN assays (Table 2). Compared with the antibody responses of sera from vaccinated monkeys at 12 weeks after vaccination, the longevity of antibody responses was confirmed. Although the antibody titers induced by the inactivated EV71 vaccine were slightly decreased, vaccinated monkeys still exhibited sufficient EV71-specific antibody responses to protect against EV71 at 54 weeks after vaccination. Notably, crossreactivity was observed against six subgenotypes throughout the duration of the experiment (Table 3). These results suggested that the inactivated EV71 vaccine could elicit strong and prolonged EV71-specific antibody responses in monkeys for more than 1 year.

**Table 2:** Humoral immune response against inactivated EV71 vaccine in monkeys.

Group		12WPV	28WPV	54WPV
**ELISA test**^a^	**EV71**	1.746±0.05***	1.80±0.04***	0.87±0.04***
	**MOCK**	0.18±0.06	0.12±0.04	0.08±0.01
**SN assay**^b^	**EV71**	20.33±0.94***	14.00±0.82***	13.67±0.47***
	**MOCK**	0	0	0

^a^ Immunized monkey sera were diluted 1:400 and then used to measure virus-specific antibodies by ELISA.

^b^ Neutralization titers are expressed as the reciprocal of the lowest dilution of antiserum that blocks infection and the mean values and SD are shown.

***There is a significant difference compared to PBS control group (*p*<0.001 tested by Two-way ANOVA test).

**Table 3:** Cross-reactivity response against inactivated EV71 vaccine in monkey.

Group	12WPV		28WPV		54WPV	
	EV71	MOCK	EV71	MOCK	EV71	MOCK
**A**	0	0	0	0	0	0
**B1**	11.67±1.25***	0	9±0.82***	0	4.67±1.25***	0
**B2**	14±0.82***	0	12.33±0.47***	0	9±0.82***	0
**B3**	12±1.41***	0	8.67±0.47***	0	7±0.82***	0
**B4**	14.33±0.94***	0	12±0.82***	0	11.33±1.25***	0
**C1**	15***	0	13.67±0.94***	0	15***	0
**C2**	2.67±0.47***	0	0	0	0	0
**C3**	0	0	0	0	0	0
**C5**	12.67±0.47***	0	8.33±0.94***	0	7***	0

Neutralization titers was assayed with various enteroviruses indicated in the left and the mean values and SD are shown.

***There is a significant difference compared to PBS control group (*p*<0.001 tested by Two-way ANOVA test).

## Discussion

Various approaches, including live-attenuated virus vaccines, inactivated virus vaccines using cell culture systems, VLP vaccines expressed by baculovirus systems in insect cells, subunit vaccines (e.g., viral antigenic protein, synthetic peptide containing epitope sites, and DNA vaccines expressed by mammalian plasmid vectors), have been used to develop an ideal EV71 vaccine to prevent HFMD [14–20]. Safety and long-lasting immunity are the most important characteristic of human vaccines. HFMD vaccines are expected to be safe in young children as vaccine recipients; this is essential because parents are particularly concerned with the safety of vaccines. Clinical trials with EV71 vaccine candidates are currently being tested in China, Taiwan, and Singapore in young adults and children [21]. Most of these studies are based on inactivated virus vaccines because of the safety of these vaccines.

HFMD vaccines against EV71 have been developed and studied in mice. Because of the difficulty of using NHPs for *in vivo* assays, efficacy tests of virus vaccines in NHPs have not been reported frequently. The efficacy of EV71 vaccine candidates was evaluated using NHPs (Taiwan macaques, rhesus macaques, and cynomolgous monkeys) in a few previous studies [15, 29–33]. Most of the efficacy studies of vaccine candidates used NHP models have evaluated inactivated virus and VLP vaccines. Moreover, the safety of inactivated EV71 vaccines has already been confirmed in NHP models and human clinical trials. In this study, the efficacy of inactivated EV71 vaccine candidates composed of a Korean isolate strain belonging to subgenotype C4a was estimated in cynomolgus monkeys. The elicited EV71-specific immune response in monkeys suggested that the vaccine candidate used in this study was safe and well tolerated in NHPs.

EV71 vaccine candidates must be safe and exhibit long-lasting immune responses. In this study, the immunogenicity and safety of an inactivated EV71 vaccine were evaluated in an NHP model for 1 year. The inactivated EV71 vaccine elicited EV71-specific immune responses and crossreactive antibody responses over the entire 54-week study. These results were consistent with previous studies in which the inactivated EV71 vaccine induced neutralizing antibodies at 33 and 56 weeks after vaccination in macaques [31, 34]. These results suggested that our vaccine candidates could induce long-lasting immune responses in an NHP model.

Due to the diversity of EV71 subgenotypes, it is important for EV71 vaccine candidates to induce crossreactive neutralizing antibody responses against heterologous strains. In this study, we demonstrated that the inactivated EV71 vaccine elicited crossreactive neutralizing antibodies against the heterologous genotypes B and C (B1, B2, B3, B4, C1, and C5). The inactivated EV71 vaccine elicited partial crossreactive neutralizing antibodies to genotype C2 but not to genotypes A and C3. There were amino acid differences between the EV71 vaccine strain and BrCr, A1451, and C3-A strains used in this study, belonging to subgenotypes A, C2, and C3, respectively. These amino acid differences could sufficiently alter conformational changes, particularly epitope sites in the VP1 region. These results suggested that EV71 vaccine strain-specific antibodies capable of crossreactive neutralizing antibodies against different genotypes of EV71 were generated by the vaccine candidate.

In summary, the inactivated EV71 vaccine derived from the C4 strain significantly induced immunogenicity in an NHP model. In particular, the inactivated EV71 vaccine was capable of inducing neutralizing antibody responses in cynomolgus monkeys and crossreactive neutralizing antibodies against heterologous strains belonging to different genotypes of EV71, with the response being maintained for 1 year. These results supported that NHPs were appropriate animal models for evaluating EV71 vaccine candidates. Further studies are needed to achieve the development of multivalent vaccines against HFMD, including diverse coxsackieviruses.

## Supporting information

S1 File The neutralizing antibody responses by a formalin-inactivated EV71 vaccine in monkey. (XLSX)
